# Epidemiologic evidence for association between adverse environmental exposures in early life and epigenetic variation: a potential link to disease susceptibility?

**DOI:** 10.1186/s13148-015-0130-0

**Published:** 2015-09-11

**Authors:** Alexander Vaiserman

**Affiliations:** Institute of Gerontology, Vyshgorodskaya st. 67, Kiev, 04114 Ukraine

**Keywords:** Developmental programming, Chronic disease, Epigenetic modification, DNA methylation, Environmental xenobiotic, Nutrition, Stress

## Abstract

A growing body of evidence suggests that the risk of development and progression of a variety of human chronic diseases depends on epigenetic modifications triggered by environmental cues during early life sensitive stages. Exposures to environmental factors such as adverse nutritional, psychological, and social conditions, as well as pollutants and substance abuse in early life, have been shown to be important determinants of epigenetic programming of chronic pathological conditions in human populations. Over the past years, it has become increasingly clear due to the epigenome-wide association studies (EWASs) that early life adverse environmental events may trigger widespread and persistent alterations in transcriptional profiling. Several candidate genes have been identified underlying these associations. In this context, DNA methylation is the most intensively studied epigenetic phenomenon. In this review, the clinical and epidemiological evidence for the role of epigenetic factors in mediating the link between early life experiences and long-term health outcomes are summarized.

## Introduction

During the past decades, the burden of chronic diseases is rapidly increasing worldwide. Adult lifestyle factors are the main risk contributors. It is, however, increasingly clear that unfavorable events during early development may also play a crucial role in the pathogenesis of chronic pathological conditions. The Developmental Origins of Health and Disease concept suggests that adverse exposures early in life may reprogram an individual for immediate adaptation to prenatal and/or neonatal environmental perturbations but enhance the risk of subsequent pathologies including cancer, type 2 diabetes (T2D), cardiovascular (CVD), and neurodegenerative disease [1].

The mechanisms implicated in developmental programming of chronic disorders are poorly understood, but epigenetic mechanisms are likely involved. In mammals, the epigenome undergoes major epigenetic modifications throughout the gametogenesis and early embryogenesis [2, 3]. In the early embryo, a dramatic reduction in methylation takes place; the methylation levels reach their minimum at the early blastocyst stage (32–64 cells). This process of epigenetic reprogramming throughout early embryogenesis erases gamete-specific methylation patterns inherited from the parents and is crucial to establishing pluripotency. After implantation, a wave of de novo methylation occurs. Another demethylation–remethylation cycle of epigenetic reprogramming takes place in the primordial germ cells which are the embryonic progenitors of oocytes and sperm. However, these processes differ in primordial germ cells and in embryos. In primordial germ cells, demethylation is close to absolute (with the exception of a few resistant retroelements), whereas in early embryos, methylation of imprinted gene regions is preserved, enabling parent-of-origin-specific gene expression in later tissues [2]. Early embryonic maintenance is especially critical in the context of developmental programming because this process is sensitive to environmental cues [3]. The epigenetic modifications triggered by environmental stimuli during these sensitive periods can persist across the life course thereby leading to pathological conditions in adulthood [4]. The role of epigenetic regulators in linking adverse early events to later outcomes is evident from both epigenome-wide association studies (EWASs) and studies of particular candidate genes.

Most data concerning developmental epigenetic programming of various pathologies have been obtained from animal models. Human findings confirming the importance of epigenetic mechanisms in such programming effects are still scarce because of limited access to relevant biological materials, but they evidently indicate that similar mechanisms may also operate in humans. This review summarizes the clinical and epidemiological evidence for the role of epigenetic factors in mediating the link between early life experiences and long-term health outcomes.

## Review

### Environmental xenobiotics

Exposure to environmental xenobiotics is among the major causes of chronic health problems in modern society. The period from preconception through weaning is likely a key time window when such exposure can harm human development [3]. During pregnancy, the environmental pollutants can cross the placenta, leading to distorted gene expression patterns and disease pathogenesis in later life [5]. Pollutants also may accumulate in breast milk to levels causing subsequent health problems, so lactation period is apparently important as well.

### Air pollution

Early life exposure to air pollution has been repeatedly shown to affect the risk of various allergic complications in subsequent life. Epigenetic modifications which are associated with inflammation and immunity are believed to be key contributors to the link between exposure to air pollution and later allergy-related conditions. In utero exposure to higher levels of traffic-related polycyclic aromatic hydrocarbons (PAHs) has been found to be associated with aberrant DNA methylation, thereby resulting in childhood asthma. For example, hypermethylation of the acyl-CoA synthetase long-chain family member 3 (*ACSL3*) gene implicated in asthma pathogenesis has been indicated in umbilical cord white blood cells of neonates who were prenatally exposed to PAHs [6].

### Endocrine-disrupting chemicals

There is a growing concern among scholars and public health officials about the risks associated with human exposure to endocrine-disrupting chemicals (EDCs), the substances which exhibit the hormone-like activity. Currently, EDCs including agricultural pesticides, dioxin, phthalates, industrial solvents, and polychlorinated biphenyls are widespread in the modern human environments [7, 8]. There is increasing evidence suggesting that early life exposure to EDCs may cause long-term health outcomes and that epigenetic mechanisms may be substantially involved. Most of these findings are derived from animal studies (for review, see [9]). Human data confirming these associations are scarce to date. The most studied EDC in the epigenetic context is bisphenol A (BPA), a carbon-based synthetic compound with estrogenic activity which is widely used in the manufacture of polycarbonate plastics and epoxy resins. There is increasing evidence that exposure to BPA in early life can promote various diseases, including infertility, metabolic disorders, and several hormone-associated tumors such as prostate and breast cancers [10]. BPA-induced alterations in the methylation of gene-coding xenobiotic metabolizing enzymes such as increased methylation at catechol-O-methyltransferase (*COMT*) and at sulfotransferase 2A1 (*SULT2A1*) promoters in human fetal liver have been revealed [11]. More recently, the global DNA methylation and expression of gene-coding metabolizing enzymes have been characterized in human fetal tissues, namely, placenta, kidney, and liver, after prenatal BPA exposure [12]. The significant tissue-specific DNA methylation differences in both long interspersed nucleotide element-1 (*LINE-1*) and CCGG content have been observed. Total BPA concentrations have been positively correlated with global methylation levels for the placenta only using the *LINE-1* assay. Expression levels of the BPA-specific metabolism genes including lysosomal enzyme beta-glucuronidase (*GUSB*), UDP glucuronosyltransferase 2 family, polypeptide B15 (*UGT2B15*), steroid sulfatase (microsomal), isozyme S (*STS*), and *SULT1A1* have been different across each tissue type.

### Heavy metals

Heavy metals including cadmium, mercury, lead, and arsenic are another important class of pollutants in modern environments [13]. Prenatal exposure to heavy metals is especially detrimental. It has been found to be a risk factor for neurological disorders and several cancers. These adverse outcomes are thought to be mediated by epigenetic changes that persist over time and affect long-term health conditions [14].

The genome-wide changes in DNA methylation caused by prenatal exposure to cadmium have been found [15]. In girls, overrepresentation of methylation changes in genes related to organ development, mineralization, and morphology of bone has been obtained, while in boys, the alterations in cell-death-linked genes have been demonstrated. In another methylome-wide association study (MWAS), prenatal cadmium exposure resulted in changed patterns of DNA methylation in leukocytes, with enrichment of genes involved in apoptosis and transcriptional regulation [16]. Periconceptional cadmium exposure has been also associated with hypomethylation of *LINE-1*, which is highly methylated in normal tissues, whereas its hypomethylation is known to cause enhanced genomic instability [17].

In some studies, DNA methylation levels in cord blood DNA of newborns have been shown to be associated with prenatal arsenic exposure [18–23]. The correlation between levels of methylation of repetitive elements such as *LINE-1* and *Arthrobacter luteus* (*Alu*, short interspersed nucleotide element), which are able to mutate other genes via their mobility, and levels of prenatal arsenic exposure has been positive among male infants but negative among female infants [19]. Mother’s urinary levels of arsenic, mostly during early pregnancy, have been associated with cord blood DNA methylation in neonates [20]. Overrepresentation of affected cancer-related genes in boys, but not in girls, has been revealed. Much weaker associations have been demonstrated with arsenic exposure in late compared to early gestation. The activation of molecular networks that are indicative of stress, inflammation, metal exposure, and apoptosis has been revealed in newborns whose mothers experienced arsenic exposure throughout pregnancy [21]. A positive correlation between methylation levels of particular loci in CpG islands in cord blood samples of infants and a mother’s urinary arsenic levels has been obtained in the Koestler et al. [22] study. Genes that demonstrated significant differences in DNA methylation after the prenatal arsenic exposure have been highly enriched for binding sites of the transcription factors such as early growth response and CCCTC-binding factors [23]. Prenatal arsenic exposure has been also associated with changes in expression of the soluble fms-like tyrosine kinase-1 (*sFLT1*) gene playing a key role in the inhibition of placental angiogenesis and related growth retardation in cord blood cells [24].

In a recent MWAS, association between DNA methylation in newborn cord blood and prenatal mercury concentrations has been revealed suggesting that exposure to mercury may lead to epigenetic modifications involved in altered immune profiles [14]. Prenatal mercury exposure has been associated with placental DNA methylation changes and adverse infant neurobehavioral outcomes in the Maccani et al. study [25]. Lead levels in maternal tissues such as patella and tibia have been shown to be inversely correlated with levels of methylation of *Alu* and *LINE-1* repetitive elements in the umbilical cord blood leukocytes of their offspring [26]. It has been also recently found using Illumina’s HumanMethylation450 BeadArray that fetal manganese exposure resulted in differential methylation of several loci in the placenta including those residing in fetal growth, neurodevelopmental, and cancer-related genes [27].

### Prenatal smoking

Maternal smoking throughout pregnancy and lactation is one of the important factors determining the process of developmental programming in modern societies. It has been repeatedly shown to cause long-term adverse health outcomes such as cognitive and behavioral impairments, T2D, CVD, respiratory illness, and cancer in offspring’s later life [28–30]. In several studies, epigenetic alterations such as changed DNA methylation and dysregulated expression of miRNA have been demonstrated to play an important role in mediating the link between maternal cigarette smoking during pregnancy and health outcomes throughout the life course [31].

Maternal smoking during pregnancy has been associated with differential DNA methylation in neonates, particularly in gene growth factor independent 1 transcription repressor (*GFI1*) implicated in diverse developmental processes and genes aryl-hydrocarbon receptor repressor (*AHRR*) and cytochrome P450 1A1 (*CYP1A1*) mediating detoxification of the components of tobacco smoke [32]. In another MWAS for maternal smoking, several CpGs in genes such as plasma membrane-associated class I myosin (*MYO1G*) and neuronal transmembrane protein member of the neurexin superfamily (*CNTNAP2*), associated with cell elasticity and migration and brain development, respectively, have been differentially methylated between the adolescents prenatally exposed or unexposed to maternal smoking [33]. In the replication cohort, the same CpGs have been differentially methylated at birth, in childhood, and throughout adolescence. Prenatal exposure to tobacco products affected both global and gene-specific DNA methylation in buccal cells of offspring during their childhood, and these effects have been modulated by variants in detoxification genes [34]. Infants born to smokers had higher levels of methylation at the differentially methylated region (DMR) of the insulin-like growth factor 2 (*IGF2*) gene in the umbilical cord blood compared to those born to never smokers or to those who quit smoking during pregnancy [35]. Prenatal smoke exposure also leads to a persistent effect on DNA methylation levels, as measured by repetitive element satellite 2 (*Sat2*), in adult peripheral blood granulocytes [36]. Methylation changes in serial blood samples at birth and at ages 7 and 17 years have been related to the duration and intensity of the maternal smoking during pregnancy [37]. In some CpG sites in genes such as Kruppel-like factor 13 (*KLF13*), phospholipid-transporting ATPase IIA (*ATP9A*), and *GFI1*, these alterations have been reversible, whereas others (*AHRR*, *MYO1G*, *CYP1A1*, and *CNTNAP2*) showed persistently distorted methylation patterns. A comparison of patterns of offspring methylation and intensity of paternal and maternal smoking demonstrated that association with maternal smoking is much stronger than with a paternal one. The role of epigenetic regulation in mediating long-lasting outcomes of maternal smoking has been observed in studying genetic pathways, such as the brain-derived neurotrophic factor (BDNF) pathway involved in brain development, and associated with the development of cognitive and affective dysfunctions. Higher rates of DNA methylation have been observed in the *BDNF-6* exon in the blood of adolescent offspring of mothers who smoked during pregnancy [38]. In utero exposure to smoking has been linked to hypomethylation of the aryl hydrocarbon receptor repressor (*AHRR*) gene in neonatal blood; further, this association has been confirmed in other cell types (buccal epithelium, cord blood mononuclear cells, and placenta tissue) from newborn twins of mothers who smoked during pregnancy [39]. Maternal smoking during pregnancy has been also related to substantial changes in DNA methylation at specific loci within the runt-related transcription factor (*RUNX3*) gene, encoding a member of the runt domain-containing family of transcription factors, in placental tissue [40]. The link between prenatal smoke exposure and expression of miRNAs has been also revealed. In the Maccani et al. study [41], maternal smoking throughout pregnancy has been related to downregulation of several miRNAs (*miR-16*, *miR-21,* and *miR-146a*) implicated in growth and developmental processes. Collectively, these findings suggest that maternal tobacco smoking during pregnancy is substantially linked to changed DNA methylation and dysregulated expression of miRNAs, but a deeper understanding of how these epigenetic alterations can affect later health outcomes remains to be elucidated [42, 43].

Evidence was also obtained suggesting that effects of maternal marijuana smoking throughout pregnancy on offspring’s health may be about the same as those of tobacco smoking [44]. A number of investigations have linked prenatal cannabis exposure to an elevated rate of intrauterine distress and growth retardation, as well as to long-term behavioral impairments and increased risk of adult neuropsychiatric disorders. These outcomes of prenatal marijuana exposure are also assumed to be related to disrupted epigenetic regulation and associated behavioral impairments [44]. Prenatal cannabis exposure has been associated with decreased expression of dopamine receptor D2 (*DRD2*) gene, which is a key element in the brain circuit controlling motivation, in the ventral striatum [45], as well as with impaired regulation of opioid-related genes [46]. These findings demonstrate that prenatal marijuana exposure causes differential regulation of several candidate genes in distinct brain circuits that may have long-term effects on cognitive and emotional behaviors.

### Prenatal alcohol exposure

Alcohol is a teratogen capable of inducing a wide range of neurobehavioral and physical abnormalities. Evidence from epidemiological studies suggest that in utero exposure to alcohol can lead to a variety of detrimental health outcomes (fetal alcohol spectrum disorders, FASDs), including impaired neuroendocrine and behavioral functioning, as well as mild cognitive impairments in later life of offspring [47]. Ethanol has been found to interfere with one-carbon metabolism and thereby with DNA methylation level, suggesting the important role of epigenetic mechanisms in the etiology of FASDs [47]. Moreover, alcohol is known to lead to a variety of nutritional irregularities such as nutrient intake, utilization, absorption, and excretion. Whereas some dietary components can substantially affect gene expression, the nutrient imbalance induced by alcohol consumption can also be the important contributor to distorted gene expression in FASD.

The role of epigenetic pathways in FASD development is evident from numerous studies reporting various fetal abnormalities and birth defects caused by preconception alcohol consumption. It suggests that alcohol exposure may induce epigenetic changes in the gametes, thereby causing the development of FASD-like phenotypes [47]. A link between chronic alcohol use and the hypomethylation of paternally imprinted loci, *H19* and *IG-DMR*, in sperm of male volunteers in genomic regions critical for embryonic development has been demonstrated [48].

Up to date, the epigenetic mechanisms of early life programming of FASD have been investigated only in a few epidemiological studies. In research by Wilhelm-Benartzi et al., *LINE-1* and *AluYb8* methylation levels in placental samples have been found to differ significantly among those infants who have been prenatally exposed to tobacco smoke and alcohol [49]. In a very recent 50 K array-based DNA methylation analysis, several identical changes in DNA methylation in the buccal epithelium of six children with FASD have been obtained, primarily in genes related to glutamatergic synapses, protocadherins and hippo signaling [50].

A summary of epidemiological findings for a potential epigenetic link between exposure to environmental xenobiotics and later risk of chronic disease is presented in Table 1.

**Table 1:** Summary of epidemiological findings for a potential epigenetic link between xenobiotic environmental exposures and chronic disease

Condition/exposure	Stage	Gene/element	Epigenetic outcome	Tissue/cells	Age at detection	Function/pathway	Ref.
PAHs	Prenatal	*ACSL3*	Hypermethylation	Umbilical cord leukocyte	Neonate	Asthma pathogenesis	[6]
BPA	Prenatal	*COMT*, *SULT2A1*	Hypermethylation	Liver	Fetal	Xenobiotic metabolism	[11]
	Prenatal	MWAS	Differential DNA methylation	Placenta, kidney, liver	Fetal	Metabolism	[12]
Cadmium	Prenatal	MWAS	Differential DNA methylation	Cord blood	4.5 years	Girls: organ development, morphology, mineralization; boys: apoptosis	[15]
	Periconception	*LINE-1*	Hypomethylation	Cord blood	Adult	Mutability	[17]
Arsenic	Early gestation	MWAS	Differential DNA methylation	Cord blood	Neonate	Boys: carcinogenesis	[20]
	Prenatal	MWAS	Differential DNA methylation	Cord blood	Neonate	Stress, apoptosis, inflammation	[21]
	Prenatal	MWAS	Differential DNA methylation	Cord blood	Neonate	Binding sites of transcription factors	[23]
Mercury	Prenatal	MWAS	Differential DNA methylation	Cord blood	Neonate	Immune system functions	[14]
	Prenatal	MWAS	Differential DNA methylation	Placenta	Neonate	Neurobehavioral pathways	[25]
Lead	Prenatal	*Alu*, *LINE-1*	Hypomethylation	Umbilical cord leukocytes	Neonate	Mutability	[26]
Manganese	Fetal	MWAS	Differential DNA methylation	Placenta	Neonate	Fetal growth, cancer, neurodevelopment	[27]
Nicotine	Prenatal	*AluYb8*, *LINE-1*	Hypomethylation	Buccal cells	Infant	Mutability	[34]
	Prenatal	*IGF2*	Hypermethylation	Umbilical cord blood	Infant	Growth, development	[35]
	Prenatal	*Sat2*	Hypomethylation	Peripheral blood granulocytes	Adult	Mutability	[36]
	Prenatal	MWAS	Differential DNA methylation	Cord blood	Neonate	Development, detoxification	[32]
	Prenatal	*MYO1G CNTNAP2*	Hypomethylation Hypermethylation	Blood	Adolescent	Cell elasticity and migration brain development	[33]
	Prenatal	*BDNF-6* exon	Hypermethylation	Blood	Adolescent	Neuronal maturation	[38]
	Prenatal	*AHRR*	Hypomethylation	Blood, placenta, peripheral blood mononuclear cells, buccal epithelium	Neonatal	Cell growth and differentiation	[39]
	Prenatal	*RUNX3*	Differential methylation	Placenta	Neonatal	Transcription factors	[40]
	Perinatal	*miR-16*, *miR-21*, *miR-146a*	Downregulation	Placenta	Neonate	Growth and developmental processes	[41]
Cannabis	Prenatal	*DRD2*	Downregulation	Ventral striatum	Fetal	Dopamine receptor	[45]
	Prenatal	Opioid-related genes	Impaired regulation	Distinct brain circuits	Fetal	Cognitive and emotional behaviors	[46]
Alcohol	Prenatal	*LINE-1*, *AluYb8*	Differential methylation	Placenta	Neonatal	Mutability	[49]
	Prenatal	MWAS	Differential methylation	Buccal cells	Childhood	Glutamatergic synapses, protocadherins, hippo signaling	[50]

## Stress

Early life exposure to stressful life events is the important risk factor for developmental programming of adverse health outcomes such as metabolic dysfunctions [51], as well as behavioral and psychiatric conditions such as anxiety, autism, and schizophrenia [52] later in life. Increasing evidence from experimental, clinical, and epidemiological studies highlight the importance of epigenetic regulation in mediating these long-term effects.

### Maternal stress and depression

Prenatal maternal stress has been repeatedly shown to be associated with deregulation of the hypothalamo-pituitary-adrenal (HPA) axis. In some studies, the crucial role of the human glucocorticoid receptor gene (nuclear receptor subfamily 3, group C, member 1, *NR3C1*) in mediating that association has been highlighted.

Enhanced methylation in the CpG-rich region of the promoter and exon *1F* of the *NR3C1* gene has been revealed in the cord blood of neonates whose mothers have been suffering from depression and anxiety during the third trimester of their pregnancy and that effect persisted through infancy up to 3 months of age [52]. Maternal depressive symptoms during pregnancy have been strongly associated with enhanced levels of *NR3C1* exon *1F* methylation in male infants and decreased methylation levels of the gene coding for another crucial factor mediating these associations, *BDNF IV*, in both male and female infants [53]. Maternal depression-associated changes in the levels of DNA methylation have been observed in neonatal T lymphocytes; those alterations persisted to adulthood in the hippocampal tissues [54].

Maternal psychological trauma during pregnancy also may significantly influence the offspring epigenome. Women’s experience of intimate partner violence during pregnancy has been revealed to be related to *NR3C1* methylation in whole-blood DNA of their adolescent offspring aged 10–19 [55]. Both the women exposed to the Tutsi genocide during pregnancy and their children had lower cortisol levels and higher methylation levels of the *NR3C1* exon *1F* than non-exposed women and their offspring [56].

### Early childhood adversity

Early postnatal period is also characterized by epigenetic plasticity. The epigenetic link between adversity in early childhood caused by exposure to emotional/psychological, sexual, and physical abuse, maltreatment, poor quality parenting or loss of parents, etc. and later-life health outcomes has been demonstrated repeatedly.

In several studies, low socioeconomic status (SES) has been used as an indicator for stressful conditions early in life. The low SES conditions have been found to be strongly associated with psycho-emotional disorders in adult life, such as schizophrenia and depression [57], as well as CVD and metabolic disorders [58]. Disadvantaged SES early in life has been related to altered whole-genome methylation profiles in blood DNA [59]. Functionally, most genes differentially methylated in association with low early life SES have been involved in various cell signaling and metabolic pathways. In another genome-wide transcriptional profiling, low-SES background in early life has been associated with significant upregulation of genes bearing response elements for the cAMP response element binding protein/activating transcription factor (CREB/ATF) family of transcription factors that conveys the adrenergic signals to leukocytes, in saliva of healthy adults [60]. Genes with response elements for glucocorticoid receptor, regulating the secretion of cortisol and transducing its anti-inflammatory actions in the immune system, were significantly down-regulated.

Moreover, those individuals who had low SES in early life also exhibited enhanced daily life cortisol secretion along with increased expression of transcript-bearing response elements for nuclear factor kappa-light-chain-enhancer of activated B cells (NF-κB) and elevated stimulated production of the pro-inflammatory cytokine IL-6. Deleterious consequences of low SES in early life on immune functions and inflammatory processes in adulthood have been shown to be buffered by high maternal warmth, and these changes have been accompanied by modulating the genome-wide transcriptional profile [61]. Low early life SES subjects whose mothers exhibited a high level of warmth toward them demonstrated reduced Toll-like receptor-stimulated production of interleukin 6 and decreased activity of both immune activating transcription factor (AP-1) and pro-inflammatory transcription factor NF-κB in adulthood compared to individuals who had low SES early in life but experienced low levels of maternal warmth.

In a genome-wide research of the long-lasting effects of unfavorable experiences such as physical and sexual abuse or neglect early in life, 362 differentially methylated promoters have been found in hippocampal neurons derived from the postmortem brain tissues of individuals with a history of severe childhood abuse relative to control subjects [62]. Genes implicated in cellular/neuronal plasticity have been most differentially methylated among abused and non-abused subjects. In another MWAS, promoters of 997 genes implicated in key cell signaling pathways related to control of transcription and development have been differentially methylated in the whole blood of adult individuals in association with their childhood abuse [63]. In a genome-wide promoter DNA methylation analysis performed in T cells from adult men, 448 gene promoters have been differentially methylated in subjects with a history of chronic physical aggression from 6 to 15 years of age relative to the control group [64]. Most of these genes are known to play an important role in aggressive behavior.

In the study by McGowan et al. [65], significant hypermethylation in the promoter and 5′ regulatory region of ribosomal RNA (*rRNA*) gene in the brain of suicidal individuals with history of early childhood neglect/abuse, consistent with decreased rRNA expression levels in the hippocampus, has been indicated relative to the control subjects. In the subsequent study, substantially reduced expression of *NR3C1 1F* has been revealed in suicide victims with a history of childhood abuse compared to non-abused suicide victims or control individuals; no difference, however, has been demonstrated between non-abused suicide victims and controls [66]. Childhood-abuse-associated decrease in *NR3C1* expression has been related to elevated methylation in the *NR3C1 1F* promoter in the hippocampus of suicide completers relative to both suicide completers with no history of abuse and control individuals [67]. Enhanced methylation of the exon *NR3C1 1F* promoter has been also found in the peripheral blood of persons with major depressive disorder and borderline personality disorder as a result of childhood maltreatment [68]. Differential DNA methylation patterns were indicated in buccal epithelium of adolescents whose parents reported high levels of stress throughout their early childhood [69]. Differential patterns of the whole-genome DNA methylation in blood samples have been evident among institutionalized children and infants raised by their own biological parents [70]. Most of differentially methylated genes have been either contributed to cell signaling pathways or to immune function, including those involved in brain development and functioning and in neural communication. One hundred seventy-three differentially methylated genes mainly implicated in inflammatory/immune responses, in several important cellular processes, and in the pathways of antigen processing and presentation, have been identified among individuals with and without previous placement into foster care [71]. A strong association has been observed between the mothers’ reports on the parenting quality provided to their children and the offspring’s methylation levels of the *NR3C1* gene and the macrophage migration inhibitory factor (*MIF*) gene involved in *NR3C1* expression and immune responses in blood samples 5 to 10 years after the assessing the caregiving quality [71]. Childhood adversity, as measured by childhood maltreatment, parental loss, and parental care, has been also linked to impaired cortisol response to stress, as well as to enhanced *NR3C1* promoter methylation in leukocyte DNA from healthy adults [72].

Overall, these data provide new insight into the mechanisms underlying early stress-induced deterioration of pathways implicated in stress reactivity and social behavior. It is assumed that early the stress-induced change of expression of the *NR3C1* gene may change the cortisol activity, thereby disturbing neuroendocrine control and impairing behavior and cognition in adulthood. There is also some evidence that unfavorable modification of epigenetic regulation triggered by stressful conditions in early life can be partly abolished via favorable psychological climate in subsequent life [61].

Table 2 summarizes epidemiological findings for a potential epigenetic link between exposure to stressful conditions in early life and later disease.

**Table 2:** Summary of epidemiological findings for a potential epigenetic link between early life stressful conditions and disease in later life

Condition/exposure	Stage	Gene/element	Epigenetic outcome	Tissue/cells	Age at detection	Function/pathway	Ref.
Maternal depression	Prenatal	*NR3C1 1F*	Hypermethylation	Blood	Neonate, infant	Stress reactivity	[52, 53]
	Prenatal	*BDNF IV*	Hypomethylation	Blood	Infant	Neuronal maturation	[53]
	Prenatal	MWAS	Differential DNA methylation	T lymphocytes, hippocampal tissues	Neonate, adult	Immune system functions	[54]
Low SES	Childhood	MWAS	Differential DNA methylation	Blood	Adult	Metabolism, cell signaling	[59]
	Childhood	*CREB/ATF* gene family *NR3C1*	Upregulation downregulation	Saliva	Adult	Inflammatory response stress reactivity	[60]
Child abuse or neglect	Childhood	MWAS	Differential DNA methylation	Hippocampal neurons	Adult	Cellular/neuronal plasticity	[62]
	Childhood	MWAS	Differential DNA methylation	Blood	Adult	Development, regulation of transcription	[63]
	Childhood	MWAS	Differential DNA methylation	T lymphocytes	Adult	Aggressive behavior	[64]
	Childhood	*rRNA* promoter	Hypermethylation	Postmortem hippocampus	Adult suicide victims	Ribosomal functioning	[65]
	Childhood	*NR3C1*	Downregulation	Postmortem hippocampus	Adult suicide victims	Stress reactivity	[66]
	Childhood	*NR3C1 1F*	Hypermethylation	Postmortem hippocampus	Adult suicide victims	Stress reactivity	[67]
	Childhood	*NR3C1 1F*	Hypermethylation	Peripheral blood	Adult	Stress reactivity	[68]
	Childhood	MWAS	Differential DNA methylation	T cells	7–14 years	Inflammatory/immune responses	[71]
	Childhood	*NR3C1*	Hypermethylation	Blood	5–10 years	Stress reactivity	[71]
	Childhood	*NR3C1* promoter	Hypermethylation	Leukocytes	Adult	Stress reactivity	[72]

## Malnutrition

Substantial research findings support the idea that inadequate nutrition early in life can be a risk factor for a variety of human diseases [73]. Important evidence for the relationship between early nutrition and adult health status comes from natural experiments, i.e., naturally occurring circumstances in which different subpopulations have different levels of exposure to supposed causal factors. Adverse long-term health outcomes have been repeatedly observed in birth cohorts exposed to famine in early life. Most well known in this aspect is research of the Dutch famine of 1944–1945. Early life undernutrition during this famine led to various adult metabolic and mental consequences including atherogenic plasma lipid profile, obesity, and CVD, as well as depression and schizophrenia [74–76]. In several studies, epigenetic differences associated with prenatal exposure to the Dutch famine have been observed. Heijmans et al. [77] identified the methylation patterns in the whole blood of siblings who have been either exposed or not exposed to the Dutch famine throughout gestation. In adult persons aged 60+, periconceptional famine exposure has been associated with decreased methylation of the *IGF2* gene involved in growth and development, in exposed compared to non-exposed siblings. In a subsequent study within the same cohort, some other genes implicated in growth and metabolic disorders, such as ATP-binding cassette transporter 1 (*ABCA1*), paternally expressed antisense transcript of the G-protein alpha subunit complex locus (*GNASAS*), insulin/insulin-like growth factor (*INSIGF*), interleukin 10 (*IL10*), leptin (*LEP*), and maternally expressed gene 3 (*MEG3*), had differential levels of DNA methylation in exposed and unexposed siblings [78]. Recently, in a MWAS conducted in the Dutch famine cohort, changes in adult whole-blood DNA methylation in persons who have been exposed to famine prenatally compared to unexposed control subjects have been revealed [79]. Early gestation, but not mid- or late gestation, has been identified as a critical period for these alterations, mostly related to genes implicated in growth, development, and metabolism. A genome-scale analysis of differential DNA methylation in the whole blood after exposure to the Dutch famine in early life revealed that prenatal malnutrition-associated differentially methylated regions (*P-DMRs*) occur preferentially at regulatory loci and map to genes enriched for differential expression throughout early development [80]. Differential methylation of the *P-DMRs* extends along pathways related to growth and metabolism, so the authors concluded that epigenetic disturbances in these pathways caused by prenatal malnutrition can provoke or exacerbate impaired metabolic phenotype later in life. Together, these data indicate that effects of prenatal famine depend on the period of exposure, with early gestation appearing to be a critical period. Since the epigenome is most sensitive to environmental cues throughout this developmental stage [3], these findings are indicative of a role of epigenetic processes in driving long-term outcomes of early life famine exposure. Epidemiological evidence for a causal association between prenatal malnutrition and adult disease is presented in Table 3.

**Table 3:** Epidemiological evidence for a causal association between prenatal malnutrition and adult disease

Condition/exposure	Stage	Gene/element	Epigenetic outcome	Tissue/cells	Age at detection	Function/pathway	Ref.
Famine	Periconceptional	*IGF2*	Hypomethylation	Blood	60+	Growth, development	[77]
	Periconceptional	*IL10*, *LEP*, *MEG*, *ABCA1*, *GNASAS*; *INSIGF*	Hypermethylation	Blood	60+	Growth, metabolic disease	[78]
			Hypomethylation				
	Early gestation	MWAS	Differential DNA methylation	Blood	Adult	Growth, development, metabolism	[79]
	Periconceptional	*P-DMRs*	Differential DNA methylation	Blood	Adult	Growth, metabolism	[80]

## Conclusions

There is increasing evidence that many human chronic disorders originate in early life, and epigenetic mechanisms play a key role in mediating these effects. Several genes have been identified as key candidate genes underlying developmental programming of particular diseases. Incorporating these novel ideas on the mechanisms of developmental epigenetic programming into the mainstream of current beliefs regarding the causation of pathological processes would certainly shift the focus of efforts targeted at preventing chronic diseases from late stages to very early stages of human life from periconception to weaning. Functional characterization of genetic pathways that are epigenetically labile in response to specific environmental cues further highlights the epigenetic mechanisms mediating the development and progression of adult-life pathologies.

Since epigenetic marks are potentially reversible, a deeper understanding of mechanisms implicated in long-term effects of early life adverse experiences will likely result in the development of new effective therapeutic options targeted to the removal of the inappropriate epigenetic marks. Current epigenetic pharmacological therapies provide clinical benefits through inhibiting DNA methyltransferases (DNMTs) or histone deacetylases (HDACs). Three HDAC inhibitors, namely, Zolinza (Vorinostat), Istodax (Romidepsin), and Beleodaq (Belinostat), as well as two DNMT inhibitors, Vidaza (5-Azacytidine) and Dacogen (Decitabine), are approved by the FDA to date [81]. Several other HDAC and DNMT inhibitors are now in development. Other promising players include non-coding RNAs such as miRNAs and long RNAs that also are known to be important modifiers of the epigenome. Although some concerns are raised because these drug candidates are unspecific and, therefore, can lead to large-scale epigenetic deregulation, there is hope that in the future more effective therapies will be developed targeting only particular epigenomic elements. Thereafter, the epigenome-targeted pharmacological therapy may likely become a powerful tool in clinical practice. Furthermore, since the early life deleterious epigenetic programming can be reversed, at least partly, by favorable psycho-social conditions later in life [61], use of self-soothing techniques, such as meditation, may be another promising therapeutic approach. Such a possibility is evident from a recent study by Kaliman et al. [82], where intensive practice of mindfulness meditation resulted in alterations of histones H4ac and H3K4me3, as well as in decreased expression of histone deacetylase genes (*HDAC* 2, 3, and 9) and pro-inflammatory genes (*RIPK2* and *COX2*) in meditators compared to control individuals. If it is possible to correct disrupted epigenetic patterns via specific epigenome-targeted therapeutic interventions, then it will be possible to prevent a variety of chronic diseases to extend the human health span.
